# Advanced, Recurrent Malignant Phyllodes Tumor With Malignant Heterologous Osteoid Differentiation Coexisting With Fibroadenoma in a 17-Year-Old Female Nigerian: A Case Report

**DOI:** 10.7759/cureus.82448

**Published:** 2025-04-17

**Authors:** Bamnan C Dallang, Kevin N Ezike, Michael E Aghahowa, Ijeoma A Okwudire-Ejeh, Emmanuel E Oguntebi, Iliya K Salu, Umar M Umar, Abdullahi Ma’aruf

**Affiliations:** 1 Anatomic Pathology and Forensic Medicine, Nile University of Nigeria, Abuja, NGA; 2 Surgery, Nile University of Nigeria, Abuja, NGA; 3 Surgery, Asokoro District Hospital, Abuja, NGA; 4 Anatomic Pathology and Forensic Medicine, Asokoro District Hospital, Abuja, NGA; 5 Radiology, Nile University of Nigeria, Abuja, NGA; 6 Obstetrics and Gynaecology, Asokoro District Hospital, Abuja, NGA

**Keywords:** adolescent female, breast, fibroadenoma, heterologous malignant osteoid, malignant phyllodes tumor

## Abstract

Fibroepithelial tumors of the breast are characterized by neoplastic proliferations of both ductal epithelial and stromal components of the breast parenchyma and exhibit biological behavior that ranges from completely benign to frankly malignant subtypes. Fibroadenoma, the prototypical benign example of these tumors, is both the most common benign tumor of the female breast overall and the most common breast tumor in adolescent and young adult females. Phyllodes tumor, on the other hand, encapsulates the full spectrum of benign to malignant biological behavior of these tumors, and while not as prevalent as fibroadenoma, it nevertheless occurs over a wider age range and is more prevalent in older females. The age incidence of phyllodes tumor also parallels its biological behavior, increasing as the spectrum progresses from benign to malignant. Fibroadenoma and phyllodes tumor share clinical and morphological characteristics, although they arise from different components of the breast parenchyma, the terminal duct lobular unit, and specialized periductal stroma. Furthermore, the malignant potential of phyllodes tumor makes its diagnostic distinction from fibroadenoma a clinical and prognostic imperative. We present the unusual case of a 17-year-old adolescent female, Nigerian, who was diagnosed with malignant phyllodes tumor of the left breast, with heterologous osteoid component, coexisting with an ipsilateral fibroadenoma, highlighting the importance of proper evaluation of breast masses regardless of patients’ age.

## Introduction

Fibroepithelial tumors of the breast are biphasic lesions characterized by proliferation of both glandular epithelial and stromal components and include tumors that exhibit morphological characteristics and biological behavior ranging from completely benign to borderline and frankly malignant subtypes [1]. The 2019 WHO classification of breast tumors names fibroadenoma, phyllodes tumor, and hamartoma as the principal fibroepithelial tumors in this category [2]. Fibroadenoma arises from the terminal duct lobular unit and is both the most common benign tumor of the female breast overall and the most common breast tumor in adolescent and young females [3,4]. Phyllodes tumor, postulated to arise from specialized periductal stroma, is characterized by a leaf-like or phyllodal pattern and accounts for about 1% of tumors of the breast, which is the commonest site of occurrence [3,5]. Phyllodes tumors are further subclassified as benign, borderline, or malignant, depending on their morphology and biological behavior [3]. The epithelial component of phyllodes tumor is usually benign; hence, the grading and prognostication are based on the histological changes observed with the stromal element [5].

Phyllodes tumors have a wide age range of occurrence, but typically occur more in older women aged between 40 and 50 years. However, a younger age of onset has been reported in Nigerians [6]. In both adults and adolescents, the vast majority of phyllodes tumors are benign, but borderline and malignant forms very rarely occur in the adolescent age group [3,5]. The typical clinical presentation is that of a painless, firm to hard, discrete mass, but it is not specific enough to distinguish it from fibroadenomas [3]. The diagnosis is therefore dependent on tissue biopsy and histology, with the key microscopic features being stromal hypercellularity and presence of an integral benign epithelial component [7].

We present the unusual case of a 17-year-old adolescent female who was diagnosed with a malignant phyllodes tumor, with heterologous osteoid component, of the left breast, coexisting with an ipsilateral fibroadenoma, highlighting the importance of proper evaluation of breast masses regardless of patients’ age.

## Case presentation

A 17-year-old, adolescent, female Nigerian presented with a history of a left breast lump of one month duration. The lump was initially painless and small (the size of a peanut) but later became painful as it progressively increased in size. It initially was not associated with nipple discharge but later became associated with yellowish nipple discharge. There was no prior history of trauma. She attained menarche at 12 years of age. There was no family history of breast lumps.

On clinical examination, an oval lump, measuring 12 x 10 cm, was palpable in the left breast and occupied the upper outer quadrant, extending to the lower outer quadrant. It was mildly tender and firm, had well-defined borders, and was not attached to underlying structures or overlying skin. There was no associated ipsilateral axillary lymphadenopathy observed.

A breast ultrasound scan detected two masses in the outer quadrants of the left breast: a huge, oval shaped, mixed echogenic complex mass, measuring 10.35 x 6.85 cm in size, involving the outer upper quadrant, showing multiple cystic portions with areas of calcifications, and exhibiting poor vascularity on Doppler interrogation; and a smaller, oval shaped, hypoechoic mass in the lower outer quadrant at about 5 o’clock position, measuring 2.35 x 1.65 cm in size. The ultrasound scan also detected enlarged axillary lymph nodes bilaterally. A sonographic assessment of a complex left breast mass (probably inflammatory) and left breast benign masses, probably fibroadenomas, was made, Breast Imaging-Reporting and Data System assessment category 2 (BI-RADS 2).

She subsequently had a WLE of the two masses. The specimens were preserved in 10% neutral buffered formalin and sent for pathological evaluation.

Grossly, the pathological examination revealed two irregularly shaped, greyish white to light tan, firm, fibrofatty masses. The margins were inked. The larger mass measured 10 x 7 x 5.5 cm and weighed 247 g, while the smaller mass measured 2.5 x 1.5 x 1.5 cm and weighed 2 g. Cut sections through the larger mass showed a partially cystic and partially solid, yellowish surface with indurated and hemorrhagic areas. The cystic areas contained dark yellow mucoid fluid. The cut section of the smaller mass showed a nodular lesion with a solid, greyish white surface, surrounded by yellowish fatty tissue.

**Figure 1:**
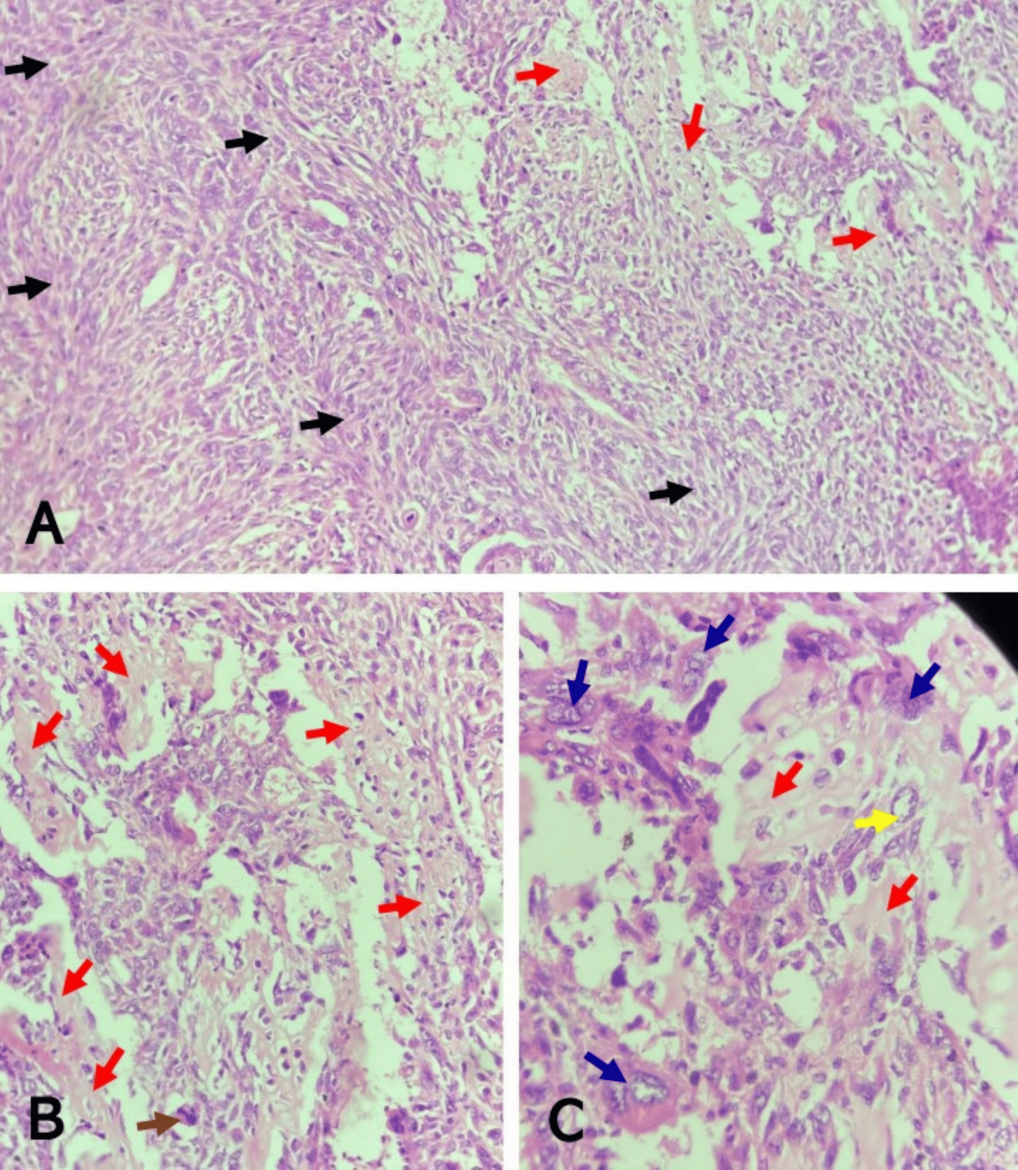
Malignant phyllodes tumor of the left breast showing malignant stroma and heterologous elements (A) Note the haphazard fascicles of malignant spindle cells (black arrows) and areas of malignant osteoid deposition (red arrows) (H&E, x100). (B) Note the malignant osteoid deposition (red arrows) with atypical osteocytes seen within lacunar spaces and abnormal mitosis (brown arrow) (H&E, x200) (C). Note the malignant osteoid deposition (red arrows), atypical osteocyte with large vesicular nucleus (yellow arrow), and rhabdoid tumor giant cells (blue arrows) seen around the osteoid (H&E, x400) H&E: hematoxylin and eosin

**Figure 2:**
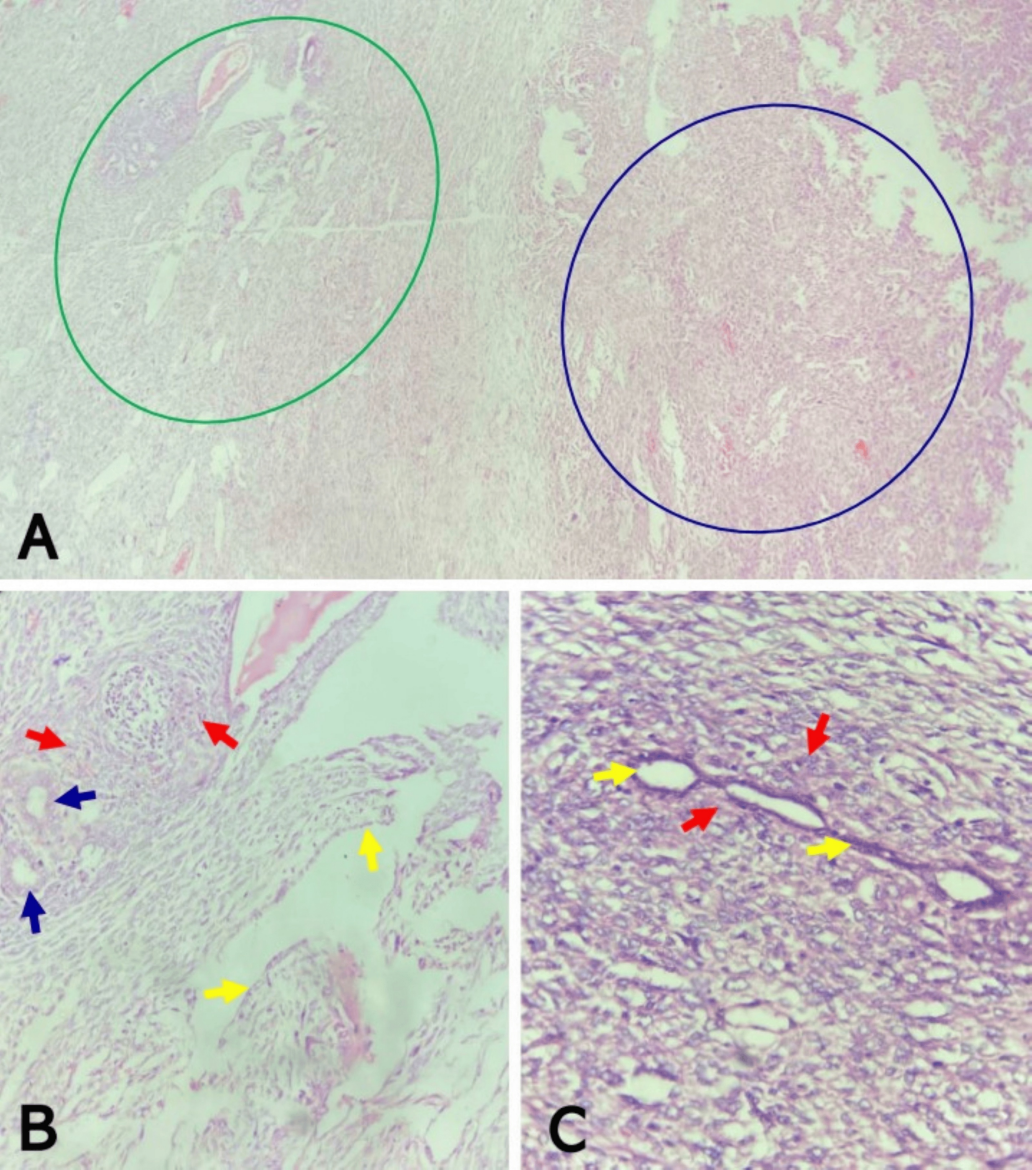
Malignant phyllodes tumor of the left breast showing epithelial elements (A) Note areas with borderline phyllodes features (green circle) adjacent to the areas of frankly malignant proliferating stroma (blue circle) (H&E, x40). (B) Note the borderline features magnified from image ‘A’, showing ducts and leaf-like structures lined by benign epithelium (blue and yellow arrows, respectively), surrounded by atypical stroma exhibiting subepithelial condensation (red arrows) (H&E, x200). (C) Note another portion of the tumor showing rare foci of benign epithelial components (yellow arrows), surrounded and compressed by proliferating malignant stroma (red arrows) (H&E, x400). H&E: hematoxylin and eosin

**Figure 3:**
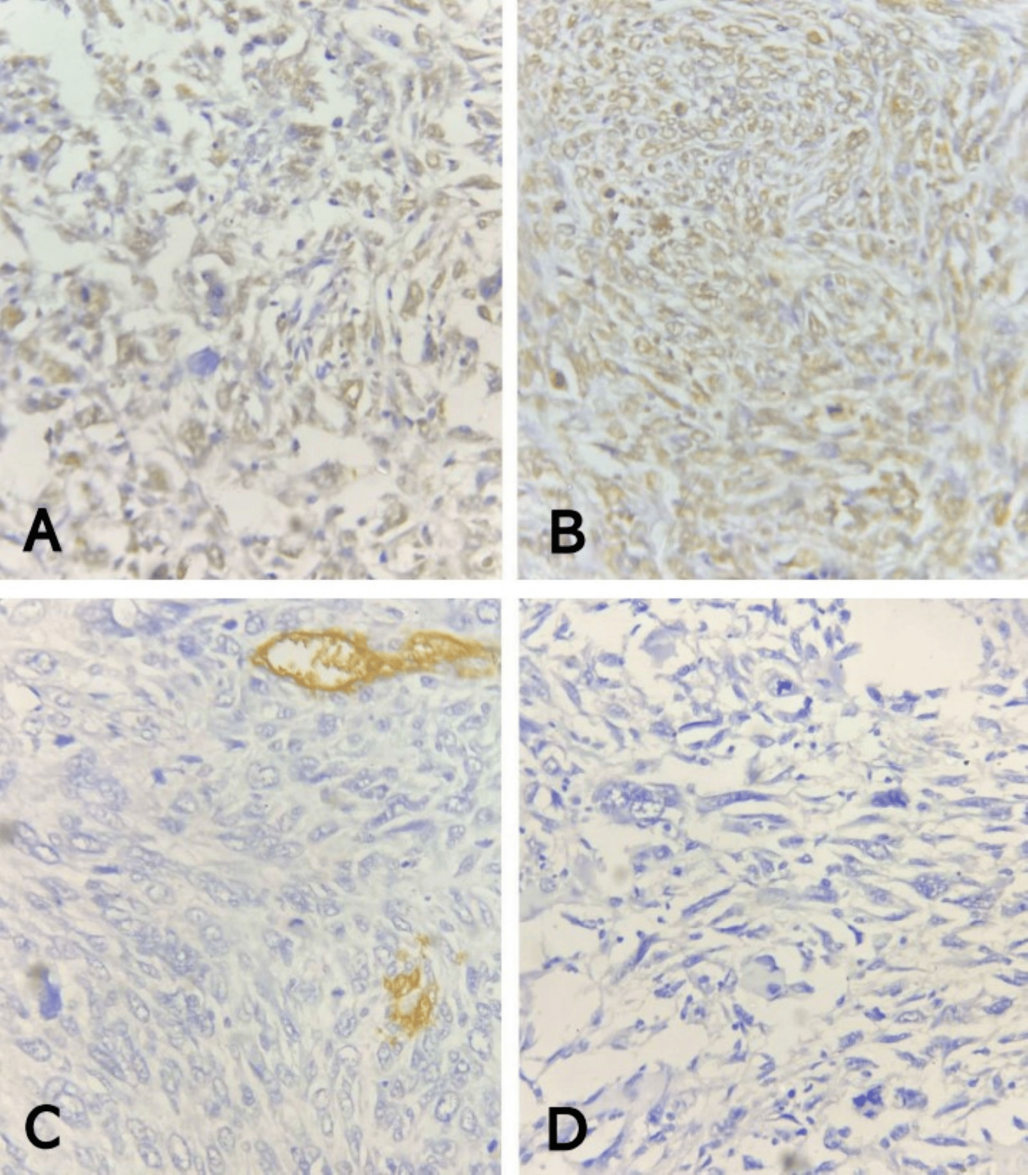
Immunohistochemistry of malignant phyllodes tumor of the left breast (A) Patchily positive P63 stain; note nuclear staining (golden brown coloration) of lesional cells (P63 IHC, x400). (B) Diffusely positive BCL2 stain; note nuclear staining (golden brown coloration) of lesional cells (BCL2 IHC, x400). (C) Negative CD34 stain; note lack of staining of lesional cells and membranous staining (golden brown coloration) outlining vascular channels (CD34 IHC, x400). (D) Negative CK5/6 stain; note lack of staining of lesional cells (CK5/6 IHC, x400) IHC: immunohistochemistry

Histological sections from the irregularly shaped mass showed a biphasic proliferation of stroma and epithelium, within effaced breast architecture, composed predominantly of high-grade, malignant sheets, whorls, fascicles, storiform, and focal herringbone patterns of atypical spindle cells set in a vascularized stroma, admixed with large, bizarre, atypical epithelioid cells. These atypical spindle cells had high-grade, round-to-oval, pleomorphic, vesicular nuclei with prominent nucleoli and fusiform cytoplasm, while the epithelioid cells were frequently rhabdoid and had high-grade, pleomorphic, vesicular nuclei and moderate-to-abundant, deeply eosinophilic cytoplasm. Mitoses were numerous, occurring >30/10HPF, and predominantly abnormal. Adjacent areas with borderline features were characterized by ducts and leaf-like structures lined by benign epithelium, surrounded by atypical stroma exhibiting subepithelial condensation. Within the frankly malignant stroma, rare foci of benign epithelial components were also seen as small ducts and slit-like structures compressed by the proliferating malignant stroma. Heterologous elements in the form of malignant osteoid were seen. Large areas of hemorrhage were also seen. The resection margins were free of tumor involvement. Immunohistochemistry showed diffuse positivity for BCL2, patchy positivity for P63, and was negative for CD34 and CK5/6 (Figures 1-3).

**Figure 4:**
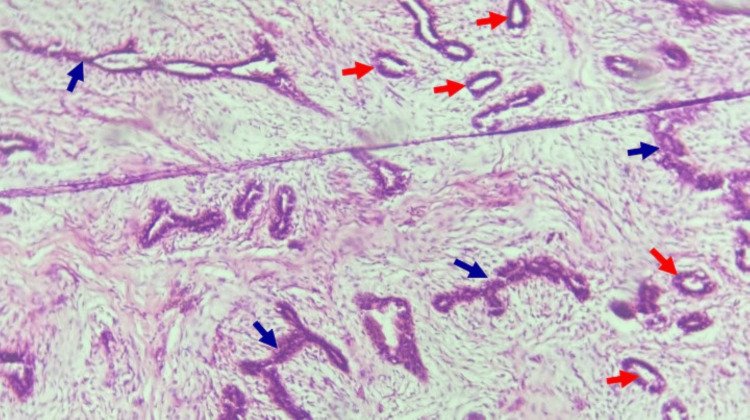
Fibroadenoma of the left breast Note mixed intracanalicular and pericanalicular patterns of the epithelial elements composed respectively of elongated and compressed slit-like structures (blue arrows) and small ducts with empty lumina (red arrows), surrounded by proliferating fibromyxoid stroma (H&E, x200) H&E: hematoxylin and eosin

Histological sections from the smaller mass showed breast tissue with distorted architecture due to an encapsulated, benign, dual proliferation of stromal and epithelial elements. The intralobular stromal component was fibromyxoid with focal areas showing hyalinization. The epithelial element showed a mixed intracanalicular and pericanalicular pattern consisting of ducts, which had been distorted into elongated and compressed slit-like structures by the proliferating stroma, and ducts with an empty open lumen. There was no cytological atypia or significant mitotic activity in any of the two components (Figure 4).

**Figure 5:**
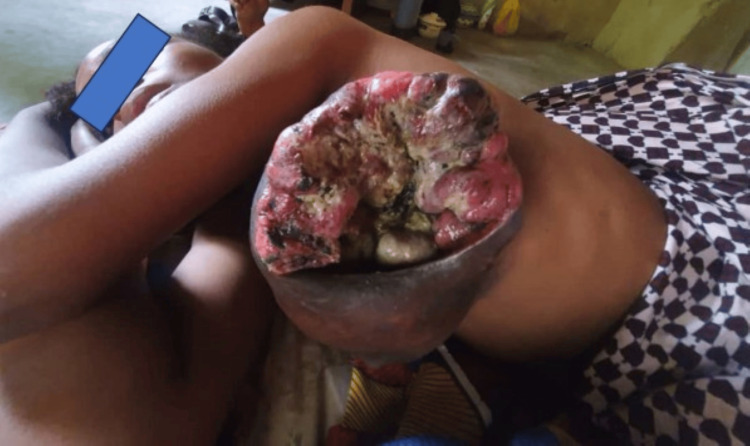
Recurrent left breast mass protruding through complete wound dehiscence

A diagnosis of malignant phyllodes with heterologous malignant osteoid, with tumor-free resection margins, coexisting with fibroadenoma was made. The patient was counselled and referred to the clinical and radiation oncologist for further management but was lost to follow-up. Four months later, she returned with complete wound dehiscence with a fungating mass, which was attached to the anterior chest wall (Figure 5).

She underwent a simple mastectomy. The mastectomy specimen was preserved in 10% neutral buffered formalin and sent for pathological evaluation.

**Figure 6:**
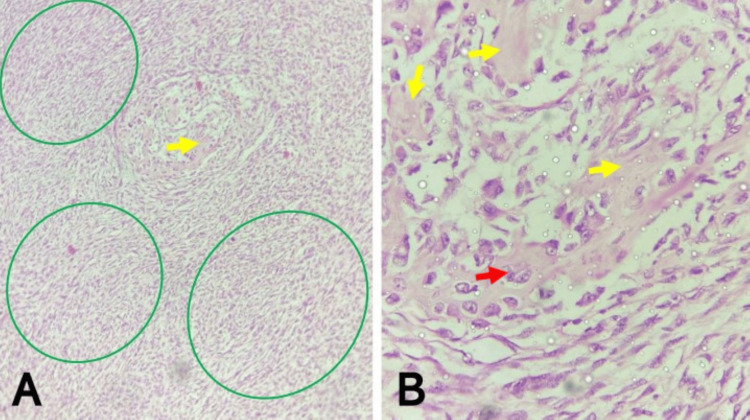
Recurrent malignant phyllodes tumor with heterologous malignant osteoid (A) Note sheets, whorls, and fascicles of malignant spindle cells (green circles), and malignant osteoid deposition (yellow arrow) (H&E, x100). (B) Note atypical osteocyte with large vesicular nucleus (red arrow), and malignant osteoid deposition (yellow arrows) within lacunar spaces (H&E x400) H&E: hematoxylin and eosin

Grossly, the pathological examination revealed a simple mastectomy specimen that weighed 1,280 g and measured 18 x 16 x 9.5 cm. A fungating greyish white nodular mass, which measured 16 cm in its widest dimension, was seen protruding through the dehiscent previous surgical incision. The margins were inked. Serial cut sections of the mass showed greyish white nodular surfaces with reddish brown areas of necrosis. The microscopic features were largely similar to the initial tumor, consisting of sheets, whorls, fascicles, storiform, and focal herringbone patterns of atypical spindle cells set in a vascularized stroma, admixed with large, bizarre, atypical epithelioid cells. The malignant osteoid was also seen. Overall, there was increased cellularity, and the deep resection margins were not free of tumor involvement. A diagnosis of recurrent malignant phyllodes tumor with heterologous malignant osteoid and tumor-positive deep resection margins was made (Figure 6).

**Figure 7:**
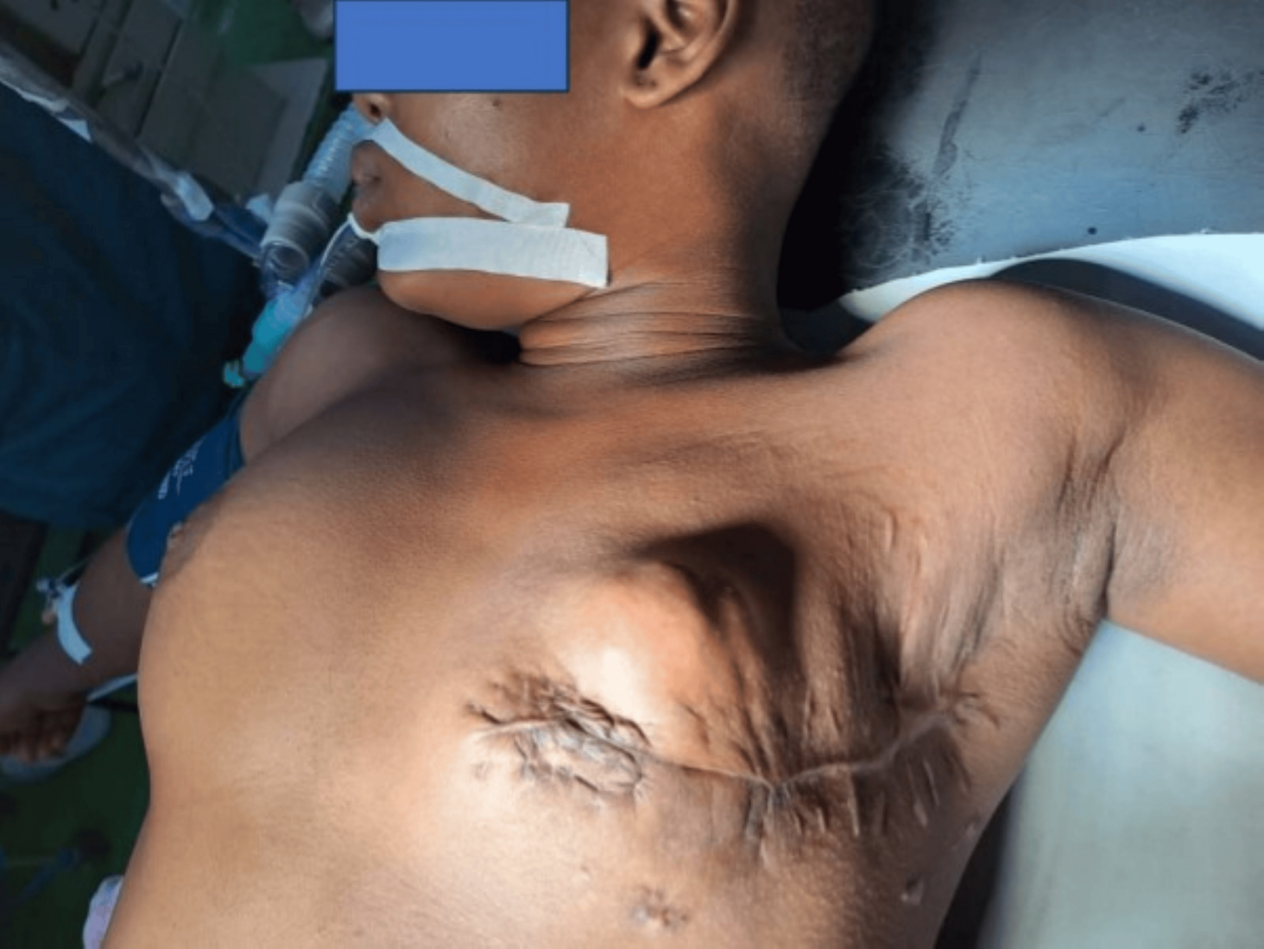
Preoperative photograph showing multiple, recurrent left chest wall nodules

The patient’s post-operative recovery was uneventful, and she was re-counselled on the importance of adjuvant chemotherapy and radiotherapy. Three months later, she returned with complaints of swellings on the operation site and left axilla and inability to pay for the prescribed oncology treatment. Clinical examination showed multiple nodular masses on the left chest wall and in the axilla. These masses were variably sized, firm, non-tender, tethered to the operation scar and underlying structures and, hence, not freely mobile (Figure 7).

She was still unable to afford oncology treatment and, on humanitarian grounds, was scheduled for re-exploration with a view to wide local excision (WLE) and left axillary node clearance (ANC). However, at operation, the chest wall recurrence was fixed to the pectoralis muscles with a central necrosis containing about 10 mL of jelly-like fluid, making WLE and ANC untenable. A local excision and axillary node sampling were done. Excised tissue was preserved in 10% neutral buffered formalin and sent for histopathological evaluation.

**Figure 8:**
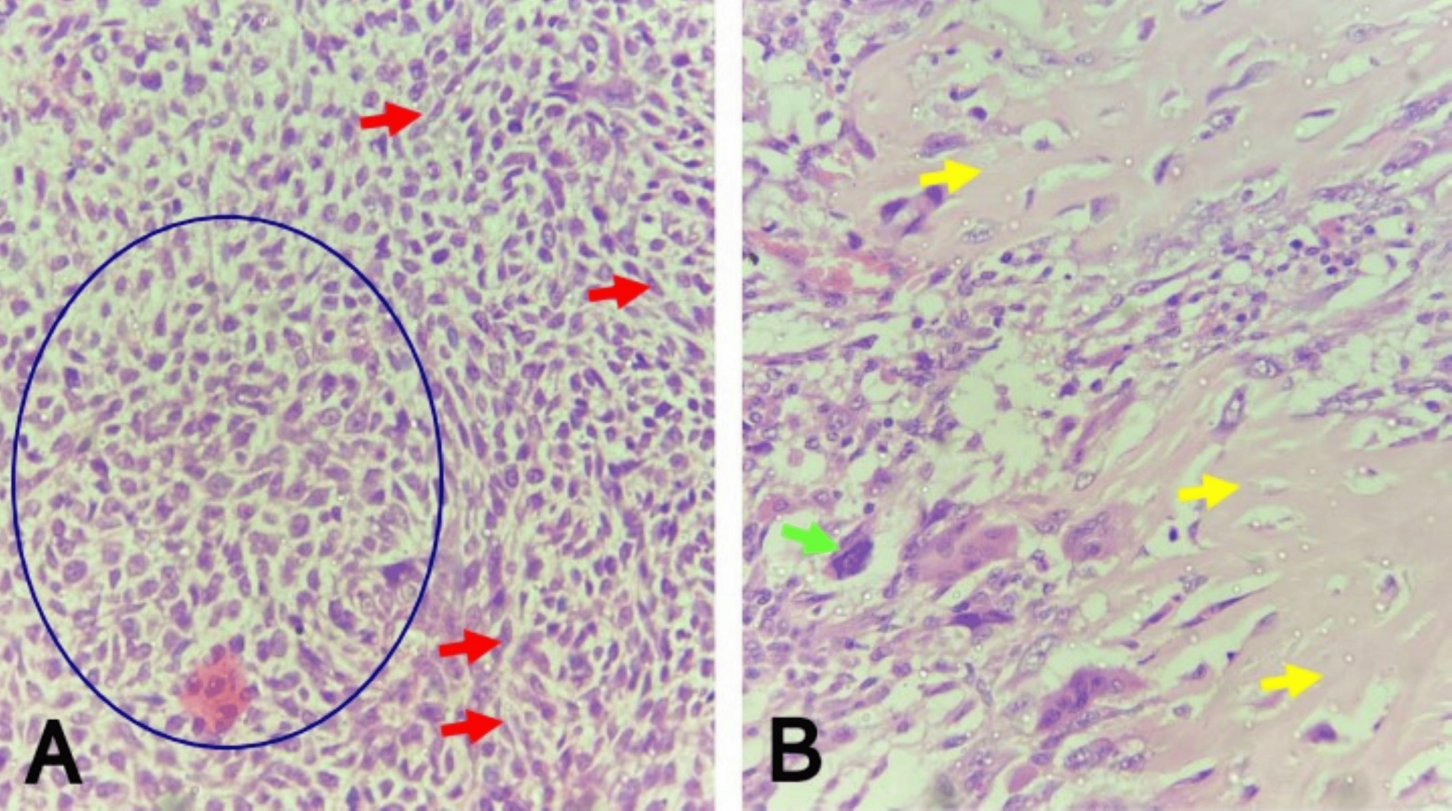
Advanced recurrent malignant phyllodes tumor with heterologous malignant osteoid (A) Note atypical spindle cells (red arrows) and sheets of atypical epithelioid cells (blue circle) (H&E, x200). (B) Note the malignant osteoid deposition (yellow arrows) and large, bizarre, tumor giant cell with enlarged hyperchromatic nuclei (green arrow) (H&E, x400). H&E: hematoxylin and eosin

Pathological examination revealed gross findings of a wedge-shaped, grayish white mass with an overlying skin and an accompanying tagged lymph node. The wedge-shaped mass weighed 34 g and measured 7 x 4.5 x 2.5 cm. The overlying skin showed areas of tethering, and a longitudinal scar that measured 7 cm long spanned the entire length of one margin of the skin. Serial cut sections showed a greyish white surface with a brownish area. The tagged lymph node weighed 7 g and measured 3 x 2 x 2 cm, and its cut section showed a greyish white surface with bony hard areas. Microscopic sections of the mass and the lymph node were similar and showed a high-grade malignant proliferation, within effaced breast and lymph node architecture, respectively, composed of sheets of epithelioid cells, and whorls, fascicles, storiform, and focal herringbone patterns of atypical spindle cells set in a vascularized stroma; and admixed with large, bizarre, tumor giant cells. These atypical spindle cells had high-grade, round-to-oval, pleomorphic, vesicular nuclei with prominent nucleoli and fusiform cytoplasm, while the epithelioid cells were frequently rhabdoid and had high-grade, pleomorphic, vesicular nuclei and moderate-to-abundant, deeply eosinophilic cytoplasm. Mitoses were numerous, occurring >30/10HPF, and frequently abnormal. There were no ductal or epithelial elements seen in the sections examined. Heterologous elements in the form of malignant osteoid were present, exhibiting increased prominence when compared to the earlier manifestations of the tumor. Areas of hemorrhage and necrosis were also seen. A diagnosis of an advanced, recurrent malignant phyllodes tumor with heterologous malignant osteoid and axillary lymph node metastasis was made (Figure 8).

Her postoperative condition was satisfactory. She was discharged on antibiotic and analgesic therapy and encouraged to source funds for her adjuvant chemoradiation. Sadly, she has been lost to follow-up.

## Discussion

Fibroadenoma and phyllodes tumors make up the vast majority of fibroepithelial lesions of the breast [8]. While fibroadenoma is common and indeed the commonest benign tumor of the breast, phyllodes tumors are relatively very rare, accounting for only 0.3%-1% of all breast tumors and 2.5% of all fibroepithelial tumors of the breast in many regions of the world [8]. Fibroadenomas are usually seen in women less than 30 years of age, but the preponderance of phyllodes tumors is seen in middle-aged women, with the average age at presentation being 40-50 years [5,8]. Malignant phyllodes tumors have a later age at presentation than their benign counterparts, occurring up to five years later [8]. Phyllodes tumors are generally less common in younger women and very rare in the pediatric age group and/or adolescents [5,8,9].

The clinical presentations of fibroadenoma and phyllodes tumors - especially the benign variant - have many similarities but also subtle differences. Both typically present as unilateral, firm, painless, mobile masses, not attached to the skin [8]. Larger lesions may show attachment to the skin with associated ulceration [5,10]. Bilaterality and multiplicity are, however, associated more with fibroadenomas [4,8]. Both lesions can be found in any part of the breast. However, while the majority of fibroadenomas are said to occur in the upper outer quadrant, phyllodes tumors can grow so big as to occupy the entire breast [11,12]. Fibroadenomas are typically slow growing and usually attain sizes of up to 3 cm or less, although larger tumors of up to 20 cm can be seen, especially in adolescents [8]. Phyllodes tumors, on the other hand, have an average size of 4-5 cm and tend to have a history of rapid increase in size and, when malignant, may be associated with tenderness and, rarely, nipple discharge [8,13]. Very large phyllodes tumors - up to 30cm - have been reported in the literature, but are relatively rare, and usually phenotypically malignant [14]. Our patient’s clinical presentation of a small left breast mass that grew rapidly to occupy more than one quadrant, became painful, and developed nipple discharge was consistent with malignant phyllodes tumor, her young age notwithstanding. Nevertheless, from the foregoing, it can be appreciated that differentiating between these fibroepithelial tumors on the basis of size alone may be difficult, particularly in adolescents. In our patient, making the distinction between the two different synchronous, ipsilateral fibroepithelial lesions was not very apparent clinically. The ultrasound scan done was, however, very useful in further raising the suspicion of the occurrence of two distinct lesions coexisting in the same breast.

The similarities in their clinical presentations belie a distinct and divergent pathogenesis. While fibroadenomas are said to arise from the terminal duct lobular unit, phyllodes tumors are postulated to arise from specialized periductal stroma [3]. The occurrence of benign phyllodes tumors with tubular adenoma-like and/or fibroadenoma-like areas arising from within the same tumor has been infrequently reported, and studies have shown limited evidence of linear progression between these two fibroepithelial lesions [15,16]. An even more unusual scenario is the coexistence of a phyllodes tumor with other ipsilateral discrete breast lesions, such as fibroadenoma, as was seen in our patient. Other studies have demonstrated the possibility of progression from benign phyllodes to higher grades of this tumor [17,18]. More studies are needed to expand and further interrogate the relationship between these two fibroepithelial lesions.

Histologically, phyllodes tumors, especially benign, and fibroadenomas share features that sometimes make distinction very difficult, thereby making their differential diagnosis from each other. Differentiating them and making an accurate diagnosis is important because of their divergent clinical progression, such as the tendency of phyllodes tumors to recur after excision and their aforementioned proclivity to transformation into more atypical forms. Important distinguishing histological features of phyllodes tumors from fibroadenoma include the increased stromal cellularity, prominent leaf-like growth pattern, and sub-epithelial stromal condensation [4]. The histological diagnosis of malignant phyllodes tumors depends on the findings of marked nuclear pleomorphism of the stromal cells, stromal overgrowth (defined as absence of epithelial elements in one low-power field containing only stroma), increased mitosis, increased stromal cellularity, and infiltrating borders [7,8]. The features of borderline tumors fall between those of the benign and malignant variants. Malignant phyllodes tumors may also exhibit malignant heterologous elements such as osteoid, liposarcomatous, rhabdomyomatous, and chondrosarcomatous, and are diagnosed as such even if the other determinants of malignancy described above are absent [8,19].

The evaluation of breast masses in general involves triple assessment: clinical assessment (history and physical examination); radiological assessment, including mammography, ultrasound, and magnetic resonance imaging (MRI); and surgical procedure, including fine needle aspiration cytology (FNAC) and core biopsy, to enable pathological diagnosis [20]. Fibroadenoma is managed either conservatively or by surgical excision and is usually not associated with recurrence [4]. Wide local excision with clear margins is the preferred treatment for benign phyllodes tumors of the breast, with adjuvant chemotherapy and radiotherapy playing limited roles in malignant subtypes whose mainstay of treatment is simple mastectomy [3,5]. Achieving negative margins of resection is invaluable in limiting local recurrence [21]. However, as our patient’s case showed, negative resection margins do not always prevent local recurrence [22,23]. Total mastectomy may also be indicated, especially in cases of local recurrence [24].

## Conclusions

The similarities in the clinical and histological features of fibroadenomas and benign phyllodes tumors make their diagnostic distinction a challenge for both surgeons and pathologists alike. However, their divergent outcomes make that distinction imperative. Furthermore, the fact that they can coexist in the same patient should encourage surgical excision in all cases of multiple breast lumps. Malignant phyllodes tumor should also be considered in the differential diagnosis of breast lumps, even in adolescents, in order to achieve early and optimal treatment.
